# A case of stuck mechanical Tricuspid Valve

**DOI:** 10.12669/pjms.40.1.8362

**Published:** 2024

**Authors:** Abdul Nasir, Mujeeb Ur Rehman, Salman Khan, Hamza Ali

**Affiliations:** 1 Dr. Abdul Nasir, MBBS, MRCS, FRCS-CTh. Consultant Cardiac Surgeon, Department of Cardiothoracic Surgery, Peshawar Institute of Cardiology, Peshawar, Pakistan; 2 Dr. Mujeeb Ur Rehman, MBBS, MS/MD, CHR. Consultant Cardiothoracic Surgeon, Department of Pediatric and Congenital Heart Surgery, Armed Forces Institute of Cardiology/National Institute of Heart Diseases (AFIC/NIHD) Rawalpindi, Pakistan; 3 Dr. Salman Khan, MS. Senior Registrar, Department of Cardiothoracic Surgery, Faisalabad Institute of Cardiology, Faisalabad, Pakistan; 4 Hamza Ali, MBBS Student. Bannu Medical College, Bannu, KPK, Pakistan

**Keywords:** Biological prosthesis, Mechanical, Tricuspid

## Abstract

A 34-year-old non hypertensive, non-diabetic and ill looking weak woman came to our emergency department with shortness of breath NYHA III-IV, severe bilateral pedal edema extending up to the thighs and gross ascites. Physical examination revealed 3mm pitting ankle and leg edema and hemodynamically was stable with raised jugular venous pressure. There was a closing and opening mechanical click on Cardiac auscultation. At the lower left sternal border, there was grade 2/6 holodiastolic rumble and a grade 2/6 systolic murmur. She had history of mitral valve replacement and tricuspid valve replacement in 2017 with mechanical valves then she had Redo tricuspid valve replacement with mechanical prosthesis again after four months. No known food or drug allergy and psychosocial issues.

Her routine bloods Labs were normal and COVID-19 was negative. On chest X-ray P/A view images and echo showed markedly gross left sided pleural effusion. In Coronary angiogram showed normal coronaries and stuck tricuspid valve (Fig.1). Echocardiography report showed preserved LV systolic function (EF=57%), dilated left atrium and right atrium. Prosthetic mitral valve was seen at mitral position, well seated and well-functioning. The mechanical mitral valve was functioning well with normal disc motion with no thrombus formation. Prosthetic tricuspid valve was seen at tricuspid level with peak gradient of 22mmHg and shown stuck mechanical tricuspid discs stuck throughout the cardiac cycle, in a fully open position (Fig.2A and 2B). Atrial fibrillation was shown on ECG. The diagnosis was made as; pannus formation resulting in mechanical TV thrombosis.

## INTRODUCTION

This patient was discussed in multidisciplinary team discussion and we planned to do Redo-tricuspid valve replacement with bio prosthesis. The patient was referred to surgery department and elective surgery was planned. Patient was taken up for a TVR with bio-prosthetic valve. Tricuspid valve was approached through right atrium by the experienced cardiac surgery team. Left atrium and Right atrium were markedly dilated and previous mechanical leaflets were stuck with thrombus and pannus formation. She underwent TVR with size 23mm Bioprosthetic Pericarbon More using 12 ×2/0 ethibond in supra-annular fashion. Procedure was uneventful and patient was shifted to the intensive care unit (ICU) in stable condition and successfully extubated after four hours. She was discharged from the hospital on 6^th^ post-operative day with stable hemodynamics, Good urine output, motion satisfactory, no ascites, no pedal edema, and no ascites.

### Follow Up

She came for follow up after one month with active complaint of generalized weakness. Her physical examination showed no ankle and thigh edema, no ascites and hemodynamically was stable with normal JVP. She came with a new look.

## DISCUSSION

TV replacement with a mechanical valve is challenging as it carries the highest risk of thrombosis with a frequency of about 3.3% of patient-years.^1^ We did not see the thrombus/pannus formation on the mitral side as there was only pannus formation on the TV mechanical valve side in our patient. No evidence based published guidelines are available for avoiding the thrombosis in patients with mechanical tricuspid valve, warfarin therapy is recommended aiming for a high therapeutic international normalized ratio value with or without supplemental antiplatelet therapy.^2^ Long term outcome of Bio prosthetic valves are reported significantly superior in patients requiring TV replacement because the chances of thrombosis is higher in mechanical valves, and structural deterioration of bio prosthetic valves have been shown.^1^

Morbidity and mortality is significantly reported in Mechanical valve thrombosis patients. Prevalence of thrombosis of right-sided valve reported significantly higher, and up to 20% of mechanical TV have been reported.^3^ Therefore, high target of international normalized ratio (INR) recommended for mechanical valves on the right side.^4^ Complications associated with the prosthetic valve on the tricuspid position, are bleeding events, valve thrombosis, and peri-valvular leakage, prosthetic valve endocarditis.^5^

In clinical practice, TVR is not that much common and is recommended in specific conditions where tricuspid valve repair is impossible or attempts to repair it have been failed.^6^ In previous studies, there is high hospital mortality reported in TVR patients, as 10.0% to 27.6%.^7^ Hence, it is been reported higher than on aortic and mitral valve replacement.^8^

Heart failure is shown the main cause of hospital mortality. Initially, Tricuspid valve disease is asymptomatic and endurable for a longer period. Most of the time, patients come for surgery with a long history of heart disease and when the right ventricular failure symptoms, such as peripheral edema ascites are shown, then the clinical and physical status of the patients is already deteriorated.^9^

Valve Thrombosis measured the Achilles’ heel of mechanical prosthesis on the tricuspid position. Its linearized rate is from 0.5% to 6.8%/ patient-year.^10^ A higher prevalence of valve thrombosis has been published in tilting disc prostheses and caged ball.^11^ it has been reported that valve thrombosis in the tricuspid position appeared in only one out of thirteen patients with the St Jude M valve with a follow-up of 13.8-year. Singh and colleagues’ results reported no thrombosis in 14 patients with follow-up of seven to ten years.^12^ Kawano and his colleagues published a retrospective study and reported that after TVR six out of nineteen SJM valves have been detected with thrombosis of valve (2.9%/patient-years), and they reported the tricuspid valve position vulnerable to thrombosis.^13^

Better bio prostheses are a good choice for low-pressure chamber (as right sided is low pressure area|) than the high pressure chamber (left sided in the mitral and aortic position).^14^ Stenosis, dysfunction and late valve calcification are the complications of bio prostheses.^15^ Bioprosthetic valves dysfunction is reported in 35% of patients with TVR after follow up for more than five years.

Long-term outcome among the two types of valves compared by Chang and his colleagues. Freedom for mechanical valves from redo-operation was 86.0% ± 6.2% at 15 years, while it was only 55.1% ± 13.8% for bio prosthetic valves. The reoperation of TVR results in high mortality and financial burden.^16^

Recently, advances in the Trans catheter valve-in-valve (VIV) technology has made the surgeons comfortable for bio prosthesis implantation. It is a charming choice to avoid redo open heart surgery.^17^ Meta-analysis reported no difference between the tricuspid mechanical and biological prosthesis. Long term survival of the mechanical valve patients has been shown in nationwide population based study.^18^

**Fig.1:**
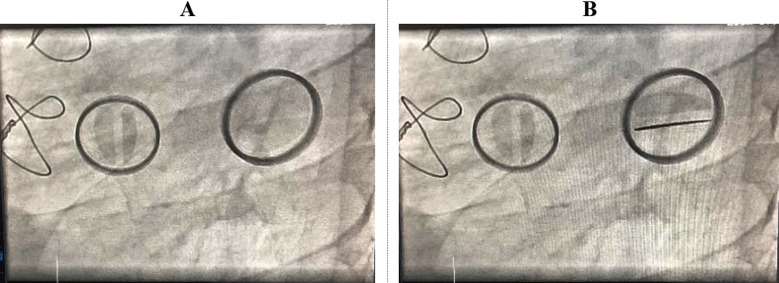
Fluoroscopy. **(A)**, Systolic and (**B**) diastolic view show that both of the discs of the mechanical tricuspid valve (TV) are stuck in an open position while mechanical mitral valve (MV) discs are closed in systole.

**Fig.2A and Fig.2B:**
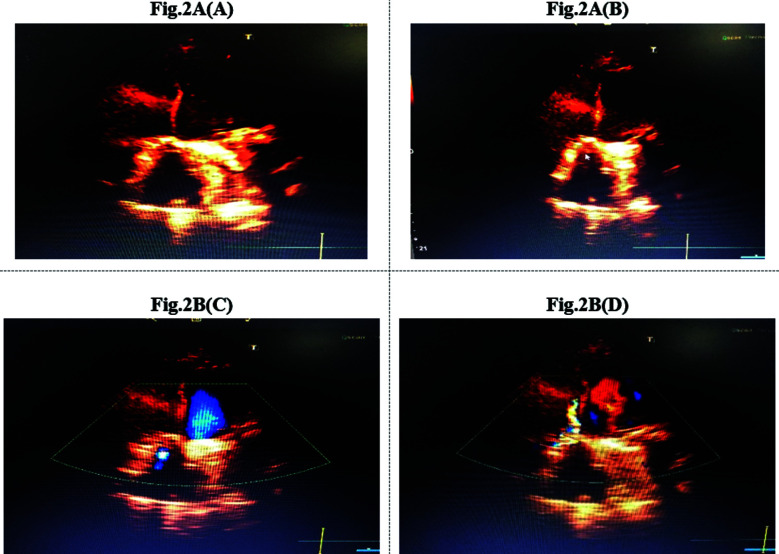
TTE of the mechanical tricuspid valve prosthesis. **(A)**, Systolic and (**B**) diastolic frames show that both discs are stuck in an open position (arrows). **(C)**, Color Doppler showing a mobile structure is intermittently noted in the medial inflow portion of the prosthesis suggestive of a Pannus/thrombus formation. **(D)**, a turbulent diastolic jet across the prosthesis.

## CONCLUSIONS

Tricuspid mechanical and bio prosthesis are having controversies and proper multi-center study is required. Our report suggested that tricuspid bioprsotheis should always be considered on the right sided low pressure area because right side is more vulnerable to thrombus formation resulting in stuck valve.

### Authors’ Contribution:

**MUR and AN:** Designed and conceived with data analysis, manuscript writing and editing.

**SK:** Helped in data collection.

**KU:** Helped in data collection, formal lay out.

**MUR and AN:** Revision, statistical analysis, and final approval.

**MUR**: Is responsible and accountable for the accuracy and integrity of the work.
